# Plasma Fibulin-3 as a Potential Biomarker for Patients with Asbestos-Related Diseases in the Han Population

**DOI:** 10.1155/2017/1725354

**Published:** 2017-10-22

**Authors:** Zhaoqiang Jiang, Shibo Ying, Wei Shen, Xianglei He, Junqiang Chen, Hailing Xia, Min Yu, Yun Xiao, Lingfang Feng, Lijin Zhu, Li Ju, Xinnian Guo, Yixiao Zhang, Jia-Wei Shen, Yan Tong, Xing Zhang, Jianlin Lou

**Affiliations:** ^1^Institute of Occupational Diseases, Zhejiang Academy of Medical Sciences, Hangzhou 310013, China; ^2^Department of Respiration Medicine, The Third People's Hospital of Cixi, Ningbo 315324, China; ^3^Department of Pathology, Zhejiang Provincial People's Hospital, Hangzhou 310014, China; ^4^School of Medicine, Hangzhou Normal University, Hangzhou 310016, China

## Abstract

Fibulin-3 has been reported as a potential biomarker for mesothelioma. However, little is known about the diagnostic efficacies of fibulin-3 for asbestos-related diseases (ARDs) in China. This study was to investigate the utility of fibulin-3 for asbestos exposure and ARDs. A total of 430 subjects were recruited from Southeast China, including healthy individuals, asbestos-exposed (AE) individuals, and patients with pleural plaques (PP), asbestosis, and malignant pleural mesothelioma (MPM). Plasma fibulin-3 was measured using the enzyme-linked immunosorbent assay. Linear regression analyses were applied to explore the influencing factors of fibulin-3. Receiver operating characteristic curves were used to determine the cutoff values. The median fibulin-3 level of subjects in the mesothelioma group was higher than that in other groups. Subjects in the asbestosis group had higher median fibulin-3 level than those in the control group. A higher fibulin-3 level was found in the group with ≥10 years of asbestos exposure as compared with control groups. The AUCs of fibulin-3 for distinguishing MPM subjects from control, AE, PP, and asbestosis subjects were 0.92, 0.88, 0.90, and 0.81, respectively. Our study provided evidence that fibulin-3 could be a potential biomarker for the early screening of MPM, but not of other nonmalignant ARDs in Chinese populations.

## 1. Introduction

Exposure to asbestos is known to result in a series of diseases called asbestos-related diseases (ARDs), including lung cancer and malignant mesothelioma, as well as nonmalignant ARDs, such as asbestosis, pleural plaques (PP), pleuritis, pleural thickening, diffuse pleural effusions, rounded atelectasis, and chronic airway obstruction [1, 2]. Based on the World Health Organization data, Diandini et al. concluded that 128,015 persons died of mesothelioma and 13,885 persons died of asbestosis from 1994 to 2010 [3]. The average potential years of life lost (PYLL) was 17 years for mesothelioma, and it was 13 years for asbestosis [3]. Pleural thickening also showed a significant association with latency time and exposure in former asbestos-exposed (AE) workers [4]. However, the long latency periods of ARDs have hampered disease prevention and precaution; most ARD patients were exposed to asbestos 20–40 years prior to disease manifestation [5]. Asbestos consumption in China has increased since the 1960s by half a million tons per year [6]. According to previous reports, China was once the top consumer and the second largest producer of asbestos in the world [7, 8]. The number of patients with ARDs is expected to rise for several decades due to the high volume of asbestos used and long latency period [6]. Thus, suitable biomarkers for ARDs are required to monitor AE workers and their families who have a history of occupational or residential exposure to asbestos.

Fibulin-3, an extracellular matrix protein, is a member of the fibulin family and a disulfide-rich glycoprotein encoded by epidermal growth factor-containing fibulin-like extracellular matrix protein 1 (EFEMP1) [9]. It is secreted by a variety of cells into the stroma and expressed in a variety of human tissues [10]. In particular, fibulin-3 may mediate cell signal transduction by bridging fiber-connecting factors and laminin in the extracellular matrix (ECM) and regulate cell proliferation in a context-specific manner. More recently, the level of tissue fibulin-3 expression has been reported to be decreased in lung cancer [11], breast cancer [12], and hepatocellular carcinoma [13]. Conversely, fibulin-3 is significantly overexpressed in pleural mesothelioma tumors compared with matched normal sites, suggesting that fibulin-3 gene expression may act as a specific biomarker for the diagnosis and prognosis of mesothelioma [14]. Furthermore, elevated circulating fibulin-3 has been reported to be one of the most intriguing molecules in the diagnosis of asbestos-induced mesothelioma [15, 16]. However, this opinion is still controversial, as it was reported that plasma levels of fibulin-3 in malignant pleural mesothelioma (MPM) patient plasma have a little diagnostic value in a European cohort but are associated with prognosis [17]. Additionally, the clinical value of circulating fibulin-3 for other nonmalignant ARDs is still unknown. Moreover, little has been done to investigate circulating fibulin-3 levels in patients with ARDs and AE population in China.

Here, we measured plasma fibulin-3 levels in the Han AE population in China and further evaluated the diagnostic value of fibulin-3 in patients with nonmalignant and malignant ARDs, including PP, asbestosis, and MPM. We provided evidence supporting the hypothesis that fibulin-3 could be a candidate biomarker in the clinical diagnosis of MPM.

## 2. Materials and Methods

### 2.1. Ethical Consideration

This study was carried out in accordance with the Declaration of Helsinki and approved by the local ethics committee of the Zhejiang Academy of Medical Sciences. The study protocol was explained to all study populations, and informed consent was provided by all study subjects.

### 2.2. Study Population and Groupings

A total of 430 subjects were recruited from a historical asbestos processing area in Southeast China. As described in Section 2.3, the subjects were divided into 5 subgroups: (1) a control group of 94 healthy individuals who were not exposed to any type of asbestos and had normal chest X-ray results, (2) an AE group of 218 individuals who were occupationally exposed to asbestos without any imaging changes, (3) a group of 74 individuals with pleural plaques (PP) alone, (4) a group of 29 patients diagnosed with asbestosis, and (5) a group of 15 patients diagnosed with MPM of epithelial type.

### 2.3. Data Collection

Data collection was carried out following previously described procedures [18]. Briefly, all subjects underwent chest X-rays. Nonmalignant ARDs were diagnosed following the American Thoracic Society (ATS) criteria [19] at the Zhejiang Academy of Medical Sciences. Blood specimens of patients with confirmed MPM were collected from two local hospitals from 2014 to 2016. Nine cases were from the Third People's Hospital of Cixi, and six cases were from Zhejiang Provincial People's Hospital. All 15 cases of MPM were confirmed as epithelial histologic subtype. The inclusion criteria was as follows: (1) MPM diagnosed by at least two independent pathologists according to the Guidelines for Pathologic Diagnosis of Malignant Mesothelioma proposed by the International Mesothelioma Interest Group [20], (2) no chemotherapy prior to blood sampling, and (3) informed consents signed by MPM patients or their lineal relatives. Those who were diagnosed with other malignant tumors were excluded from our study. A venous blood sample was obtained from all participants at the time of diagnosis before receiving any treatment.

A standardized questionnaire focusing on occupational history was taken during a face-to-face interview. The questionnaires collected information such as age, gender, smoking and drinking habit, occupation, and previous jobs entailing exposure to asbestos. For those who were occupationally exposed to asbestos, the exposure duration was measured as the interval of years between the start and the end of jobs entailing exposure to asbestos. Potential exposure levels to asbestos were estimated by the product of median airborne asbestos concentration and asbestos exposure duration, according to the approach in our previous report [21]. Briefly, the data of total dust concentrations was retrieved in different asbestos processing plants available from 1984 to 2010 in this area. The median dust concentrations were 0.7 mg/m^3^ in small-scale household workshops and 5.9 mg/m^3^ in large-scale manufactories. Based on the corresponding median dust concentration and the records of asbestos exposure duration, potential exposure levels of subjects were calculated as the cumulative sum of products of asbestos fiber dust concentration and asbestos exposure duration in each worksite (mg/m^3^ × years).

### 2.4. Detection of Fibulin-3

Five milliliters of blood samples with ethylene diamine tetraacetic acid anticoagulation was collected from all study participants. These samples were centrifuged at 2,500 rpm for 10 min. Then, the plasma was transferred into Eppendorf tubes and stored at −80°C for further detection. Fibulin-3 was determined with a two-stage enzyme-linked immunosorbent assay (ELISA) kit (Cloud-Clone Corp., Houston, TX, USA) according to the manufacturer's protocol. Briefly, plasma samples were directly tested without dilution, and all tests were performed in duplicate. The readouts and standard curves were performed using a SpectraMax M4 multimode microplate reader (Molecular Devices, Sunnyvale, CA, USA) and measured at 450 nm immediately. The results of fibulin-3 levels were calculated and visualized as ng/ml for statistical analysis.

### 2.5. Statistical Analysis

Qualitative data were reported as frequency and proportion distributions. Chi-square tests were used to compare the proportions between groups, and the significance level was adjusted by the Bonferroni method. Quantitative data with nonnormal distribution were presented as a median (interquartile range). The Kruskal-Wallis test followed by Dunn's multiple comparison test was conducted to compare the differences in the median values between groups. Univariate and multivariate linear regression analyses were fitted to explore the predictors of fibulin-3. Receiver operating characteristic (ROC) curves were analyzed to differentiate the ARD and AE groups from the control group. The best statistical cutoff value was selected when Youden's index (sensitivity + specificity − 1) reached the maximum value. The area under the ROC curves was reported with 95% confidence intervals (CIs). An area under the ROC curve of 1.0 indicates perfect discrimination, whereas an area with a confidence interval of 0.5 indicates that the discriminatory ability of the test was not better than chance. A two-sided *P* < 0.05 was considered to be statistically significant. Analyses were performed with GraphPad Prism 5.0 and SPSS Statistics 17.0 (SPSS Inc., Chicago, IL, USA).

## 3. Results

### 3.1. General Characteristics of the Study Groups

The general and occupational characteristics of the subjects, including age, gender, smoking status, drinking status, asbestos exposure, potential exposure level of asbestos, and exposure duration, are presented in Table 1. The age between these groups was significantly different (*χ*^2^ = 13.68, *P* = 0.01). Subjects with asbestosis were significantly older (median age, 73 years) than the subjects in the MPM (median age, 66 years; *P* < 0.05) and AE (median age, 67 years; *P* < 0.05) groups. The distribution of gender was significantly different among the groups (*χ*^2^ = 38.63, *P* < 0.01), and the PP, AE, and asbestosis groups had a larger proportion of females compared with the control group (*χ*^2^ = 7.62, 18.72, and 11.66; *P* = 0.01, *P* < 0.01, and *P* < 0.01, resp.). The distribution of smoking and drinking habits was significantly different among the groups (*χ*^2^ = 11.98 and 12.81; *P* = 0.02 and 0.01, resp.). In contrast, no significant difference was observed with regard to exposure duration and the potential exposure level (*P* = 0.24 and 0.25, resp.).

### 3.2. ARDs Have a Significantly Positive Influence on Fibulin-3 Levels

To test whether the predicted factors, such as age, gender, smoking habit, drinking habit, and study groups, influence plasma levels of fibulin-3, we carried out univariable and multiple linear regression analyses. Data regarding the regression coefficients, standard error of the coefficients, and *P* values analyzed by linear regression are shown in Table 2. The results of the univariable regression analysis indicated that neither gender nor smoking habits have significant influence on plasma levels of fibulin-3. However, the dummy variables of the PP, AE, asbestosis, and MPM groups exhibited a significant influence on fibulin-3 levels with a positive coefficient in both the univariable regression analysis and multivariable regression analysis.

### 3.3. Elevated Fibulin-3 Levels in the ARD Groups

**Figure 1:**
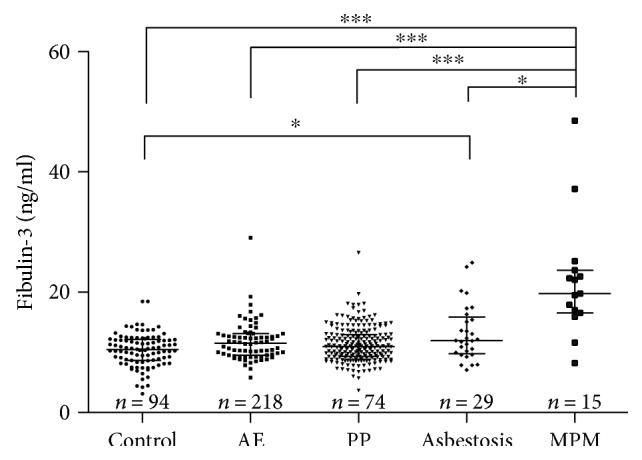
Plasma fibulin-3 levels in individuals with pleural plaques (PP), asbestosis, malignant pleural mesothelioma (MPM), and asbestos exposure (AE) and healthy controls (control). ELISA as shown were performed in parallel and blindly. Bars represent median with interquartile 25–75. Statistical significance was defined as two-sided ^∗^*P* < 0.05 and ^∗∗∗^*P* < 0.001.

To investigate whether fibulin-3 functions as a potential biomarker for identifying ARD individuals in the Han population, we tested the levels of fibulin-3 in plasma from all five groups. There was a significant difference in fibulin-3 expression among the groups (*χ*^2^ = 37.63, *P* < 0.0001; Figure 1). The results of multiple comparison analysis showed that fibulin-3 levels of subjects in the MPM group (median level: 19.8 (7.1) ng/ml) were higher than those in the asbestosis group (median level: 12.0 (5.5) ng/ml), the PP group (median level: 11.5 (3.6) ng/ml), the AE group (median level: 11.0 (3.6) ng/ml), and the control group (median level: 10.5 (3.5) ng/ml). Subjects in the asbestosis group exhibited higher fibulin-3 levels than controls (*P* < 0.05). However, fibulin-3 levels between the other groups were not significantly different (*P* > 0.05).

### 3.4. Higher Levels of Fibulin-3 in Subjects with Longer Exposure Duration

**Figure 2:**
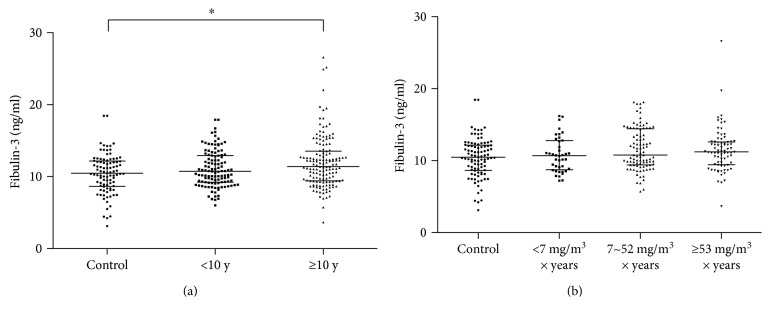
Plasma fibulin-3 levels of subgroups within AE subjects. The data on the subgroup of different exposure duration (a) and different potential exposure level (b) are shown, respectively. Dark line, median; bars represent median with interquartile 25–75. y: years. Statistical significance was defined as two-sided ^∗^*P* < 0.05.

To further ascertain whether the plasma level of fibulin-3 is associated with asbestos exposure duration and potential exposure level, we compared fibulin-3 levels among control and AE groups. Interestingly, there was a significant difference among different subgroups of exposure duration within the AE group (*χ*^2^ = 10.19, *P* = 0.001), as shown in Figure 2. In detail, AE subjects with ≥10 years of exposure had higher levels of fibulin-3 (median value: 11.4 (4.1) ng/ml) than the control group (median value: 10.5 (3.5) ng/ml). However, no significant difference was found among groups with different levels of potential exposure compared to the control group (*χ*^2^ = 7.31, *P* = 0.06; Figure 2).

### 3.5. Plasma Fibulin-3 as a Diagnostic Biomarker for MPM

**Figure 3:**
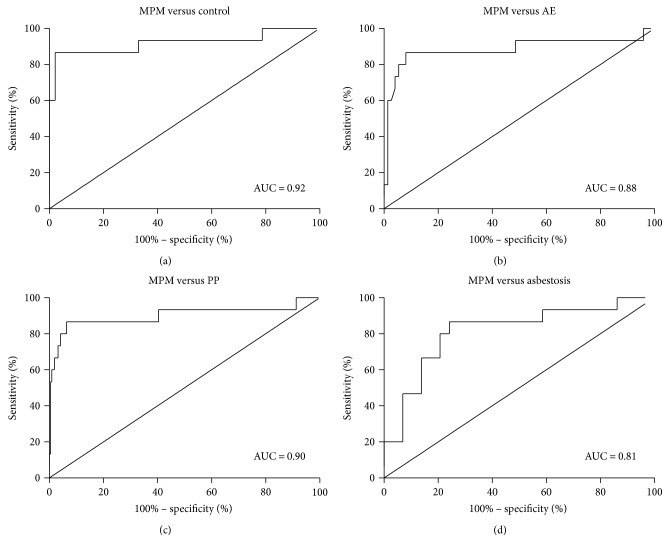
ROC curves of plasma fibulin-3 for distinguishing patients with asbestos-related diseases (ARDs) from AE subjects or healthy controls. Four ROCs with high AUC (>0.800) were selected and are shown in (a), (b), (c), and (d). ROC: receiver operating characteristic; AE: asbestos-exposed subjects; AUC: area under the curve.

To further evaluate the diagnostic value of fibulin-3 in differentiating ARD patients from AE individuals and healthy controls, we calculated the sensitivity and specificity of fibulin-3 as a potential biomarker (Table 3). The area under the curve (AUC) for fibulin-3 was 0.92 (95% CI: 0.81–1.00) for distinguishing MPM subjects from control subjects (Figure 3(a)). A fibulin-3 level of 15.30 ng/ml was determined to be the most optimal cutoff value, with a sensitivity of 86.67% and a specificity of 97.87% for the differentiation of MPM patients and controls (Figures 3(b) and 3(d)). The AUCs for fibulin-3 values for distinguishing MPM subjects from AE, PP, and asbestosis subjects were 0.88 (95% CI: 0.75–1.00), 0.90 (95% CI: 0.78–1.00), and 0.81 (95% CI: 0.67–0.96), respectively. These results suggest that plasma fibulin-3 could serve as a useful biomarker for the diagnosis of MPM. The best cutoff value was 15.84 ng/ml (sensitivity: 86.67%, specificity: 91.89%) for distinguishing MPM subjects from AE subjects, 15.86 ng/ml (sensitivity: 86.67%, specificity: 93.58%) for distinguishing MPM subjects from PP subjects, and 15.69 ng/ml (sensitivity: 86.67%, specificity: 75.86%) for distinguishing MPM subjects from asbestosis subjects. The AUCs of fibulin-3 for distinguishing AE, PP, and asbestosis subjects from subjects of the control group were 0.61 (95% CI: 0.53–0.70), 0.57 (95% CI: 0.50–0.64), and 0.67 (95% CI: 0.55–0.79), respectively.

To summarize, the fibulin-3 possesses a high diagnostic value for distinguishing MPM subjects from AE, PP, and asbestosis subjects, respectively.

## 4. Discussion

In this study, we found that elevated plasma levels of fibulin-3 could differentiate MPM and asbestosis patients from healthy controls. Our results were supported by a previous study which reported that plasma fibulin-3 levels were highly increased in mesothelioma patients compared to AE individuals and healthy controls [16]. A fibulin-3 level of 15.30 ng/ml was determined to be the best cutoff value for the diagnosis of MPM, with a high sensitivity of 86.67% and a high specificity of 97.87%. However, fibulin-3 is unlikely to be a useful marker for screening asbestosis due to a low AUC of 0.67. Thus, we propose that fibulin-3 could be a valuable indicator in the identification of individuals who have developed malignant mesothelioma.

ARDs are believed to be caused mainly by long-term exposure to asbestos fibers or erionite [22]. The number of ARD cases is rising worldwide due to asbestos exposure in the 1960s–1980s [23]. Occupational or environmental asbestos exposure could lead to malignant ARDs, such as mesothelioma or lung cancer. However, early diagnosis of these malignant diseases, in particular MPM, is usually difficult due to the lack of an effective diagnostic method. Hence, the search for new biomarkers is considered a very promising field. Several molecules reflecting changes in biologic function in response to asbestos exposure have been evaluated for their utility as biomarkers [24, 25]. For example, HMGB1 was thought to be a potential indicator for screening severe ARDs and evaluating high-risk AE cohorts in our previous work [18]. Soluble mesothelin-related protein [26], osteopontin [27], and megakaryocyte potentiating factor [28] were also regarded as helpful biomarkers for the diagnosis of mesothelioma or the prediction of the risk of mesothelioma in AE individuals. However, useful biomarkers for the diagnosis of ARDs are still lacking.

Of particular interest is the possibility that fibulin-3 may become a blood biomarker for malignant mesothelioma or other ARDs. Furthermore, the clinical value of circulating fibulin-3 in the nonmalignant ARD and AE populations is still unclear. To our knowledge, this is the first study to report the utility of plasma fibulin-3 in differentiating individuals with malignant mesothelioma from AE individuals and individuals with benign ARDs, such as asbestosis and PP, in the Han population. A recent study found no difference in plasma fibulin-3 levels between patients with asbestosis and controls [29]. But our data showed elevated fibulin-3 levels in the asbestosis group compared to the control group. However, the relatively low AUC of 0.67 for fibulin-3 may limit its epidemiological usefulness for identifying asbestosis patients among large cohorts in the asbestos processing area. From our perspective, the combination of fibulin-3 and other biomarkers with high sensitivity would result in an even greater discriminatory ability. Furthermore, previous results indicated that plasma fibulin-3 levels between the control group and AE group were not significantly different [16]. Nevertheless, we found that plasma fibulin-3 levels were significantly elevated in subjects with ≥10 years of exposure compared to healthy controls, which indicated a possible time-response relationship between asbestos exposure and plasma fibulin-3. Thus, our results suggested that an increase in plasma fibulin-3 may occur as a consequence of continuous exposure to asbestos and that fibulin-3 could be a desirable biomarker for discriminating subjects with a longer duration of exposure. As the exposure duration of asbestos was reported to be highly correlated with the development of mesothelioma [30], it can be inferred that high plasma fibulin-3 levels in subjects with a long exposure duration may be interpreted as suggestive of MPM.

Currently, fibulin-3 has become one of the most intriguing molecules in tumor development and progression. For example, increased fibulin-3 expression was found to inhibit TGF-*β*-induced epithelial-mesenchymal transition (EMT) and endothelial permeability as well as cell morphology, growth, invasion, adhesion, and migration in breast cancer [31, 32], whereas the loss of fibulin-3 expression/function promoted these TGF-*β*-mediated effects [33]. Overexpression of fibulin-3 suppressed the invasion and migration of lung adenocarcinoma cells and suppressed the expression of N-cadherin and Snail, which are regarded as EMT activators [34]. Fibulin-3 was also reported to inhibit extracellular signal-regulated kinase, leading to the activation of glycogen synthase kinase 3*β* and the suppression of Wnt/*β*-catenin signaling [35] as well as the inactivation of the upstream regulators of GSK3*β*, such as phosphoinositide 3-kinase and insulin-like growth factor-1 receptor. According to the results of gene expression analysis, fibulin-3 was significantly overexpressed in pleural mesothelioma tumors compared with matched normal sites, which suggested that fibulin-3 gene expression may act as a predictive biomarker for the prognosis of surgical intervention [14]. Combining these results with our findings, it is likely that intracellular fibulin-3 overexpression undergoing the sustained stimulation of asbestos fibers may induce tumor development and promote its sections into the blood. However, the underlying mechanism of fibulin-3 release and circulation in ARDs remains to be elucidated in future studies.

Our current data confirmed the value of fibulin-3 for the diagnosis of MPM and verified that fibulin-3 could serve as a biomarker for the differentiation between nonmalignant ARD patients and healthy subjects. In our results, the median level of plasma fibulin-3 from MPM patients is lower than that previously reported [16, 36] but is higher than or relatively close to that in other recent published articles [17, 29, 37]. According to the discrepancies between this study and some past reports, we suspect that the level of plasma fibulin-3 levels does vary according to the cohort from different races. Of course, the storage time of blood samples and experimental conditions could also be considered possible influencing factors. In addition, the statistical results indicated by the mean [16, 17] should be different from those indicated by the median [37]. The limitation was that fibulin-3 was detected in a limited number of MPM patients, as nationwide data on mesothelioma are scarce in China due to the lack of a nationwide registry of mesothelioma patients [38]. Moreover, published data on Chinese MPM patients remain insufficient, and a follow-up study is needed to validate the clinical value of fibulin-3 in the early diagnosis and screening of mesothelioma. Of course, more cases of mesothelioma should be included in future studies in order to increase the statistical power and broaden the clinical research.

## 5. Conclusions

The present study aimed to assess the relationship between plasma fibulin-3 levels and previous asbestos exposure and the utility of fibulin-3 in the detection of ARDs. Our findings indicated that plasma fibulin-3 is elevated in MPM and asbestosis patients. Notably, fibulin-3 may possess a diagnostic value for differentiating MPM case from cases of AE, PP, and asbestosis. Hence, our study provides supporting evidence that fibulin-3 could be used as a potential biomarker for the early screening in high-risk AE and healthy Chinese populations for MPM, but not for other nonmalignant ARDs.

## Figures and Tables

**Table 1:** Basic characteristics of the subjects.

	Control (*n* = 94)	AE (*n* = 218)	PP (*n* = 74)	Asbestosis (*n* = 29)	MPM (*n* = 15)
Age, y (median (Q1–Q3))	67 (64–73)	67 (62–72)	69 (63–74)	73 (65–82)	66 (57–72)
Gender					
Male, *n* (%)	57 (61)	54 (25)	29 (39)	8 (28)	7 (47)
Female, *n* (%)	37 (39)	164 (75)	45 (61)	21 (72)	8 (53)
Smoker, *n* (%)	14 (16)	17 (8)	13 (19)	1 (4)	4 (27)
Drinker, *n* (%)	8 (9)	11 (5)	7 (10)	0 (0)	4 (27)
Exposure to asbestos (yes/no)	0/94	218/0	48/26	23/6	8/7
Potential exposure level, mg/m^3^ × years (median (Q1–Q3))	NA	35 (7–77)	30 (4–59)	30 (7–100)	11 (4–19)
Exposure duration, y (median (Q1–Q3))	NA	9 (5–14)	10 (5–14)	11 (6–17)	14 (5–19)

PP: pleural plaque; AE: asbestos-exposed subjects; MPM: malignant pleural mesothelioma; Q1: 1st quartile; Q3: 3rd quartile; y: years; NA: not applicable.

**Table 2:** Factors influencing plasma levels of fibulin-3 in univariate and multivariate linear regression models.

Predictor variables^∗^	Subcategory	Univariate models			Multivariate model^#^		
		b	SE	*P* value	b	SE	*P* value
Age, y	—	<0.01	<0.01	0.97			
Female versus male	—	0.03	0.03	0.30			
Nonsmoker versus smoker	—	−0.03	0.05	0.59			
Nondrinker versus drinker	—	0.01	0.06	0.89			
Study groups	PP versus control group	0.13	0.04	<0.001	0.13	0.04	0.002
	AE versus control group	0.09	0.03	0.01	0.09	0.03	0.01
	Asbestosis versus control group	0.22	0.06	<0.001	0.22	0.06	<0.001
	MPM versus control group	0.70	0.08	<0.001	0.70	0.08	<0.001

^∗^The dependent variable was the natural logarithm form of fibulin-3; ^#^estimated with the forward stepwise method; *b*: regression coefficient; y: years; PP: pleural plaque; MPM: malignant pleural mesothelioma; AE: asbestos-exposed subjects; SE: standard error.

**Table 3:** AUC and cutoff value of plasma fibulin-3 for diagnosing ARDs.

	AUC (95% CI)	Cutoff value (ng/ml)	Sensitivity (%)	Specificity (%)	*P* value
MPM versus control	0.92 (0.81–1.00)	15.30	86.67	97.87	<0.001
MPM versus AE	0.88 (0.75–1.00)	15.84	86.67	91.89	<0.001
MPM versus PP	0.90 (0.78–1.00)	15.86	86.67	93.58	<0.001
MPM versus asbestosis	0.81 (0.67–0.96)	15.69	86.67	75.86	0.001
Asbestosis versus controls	0.67 (0.55–0.79)	14.88	31.03	97.87	0.007
AE versus controls	0.61 (0.53–0.70)	12.26	41.89	77.66	0.014
PP versus controls	0.57 (0.50–0.64)	8.40	90.83	23.40	0.040

AUC: area under the curve; CI: confidence interval; PP: pleural plaque; MPM: malignant pleural mesothelioma; AE: asbestos-exposed subjects; ARDs: asbestos-related diseases.

## Abbreviations

ARDs: Asbestos-related diseases
AUC: Area under curve
CI: Confidence interval
Q1: 1st quartile
Q3: 3rd quartile
MPM: Malignant pleural mesothelioma
PP: Pleural plaque
ROC: Receiver operating characteristic.

## References

[1] WHO (1998). *Environmental Health Criteria 203: Chrysotile Asbestos*.

[2] Harbut M. R. (2007). Treatment of nonmalignant asbestos-related diseases. *American Journal of Industrial Medicine*.

[3] Diandini R., Takahashi K., Park E. K. (2013). Potential years of life lost (PYLL) caused by asbestos-related diseases in the world. *American Journal of Industrial Medicine*.

[4] Algranti E., Mendonca E. M., DeCapitani E. M., Freitas J. B., Silva H. C., Bussacos M. A. (2001). Non-malignant asbestos-related diseases in Brazilian asbestos-cement workers. *American Journal of Industrial Medicine*.

[5] Mesaros C., Worth A. J., Snyder N. W. (2015). Bioanalytical techniques for detecting biomarkers of response to human asbestos exposure. *Bioanalysis*.

[6] Courtice M. N., Lin S., Wang X. (2012). An updated review on asbestos and related diseases in China. *International Journal of Occupational and Environmental Health*.

[7] Haynes R. C. (2010). A worn-out welcome: renewed call for a global ban on asbestos. *Environmental Health Perspectives*.

[8] Wang X., Yano E., Lin S. (2013). Cancer mortality in Chinese chrysotile asbestos miners: exposure-response relationships. *PLoS One*.

[9] Panou V., Vyberg M., Weinreich U. M., Meristoudis C., Falkmer U. G., Roe O. D. (2015). The established and future biomarkers of malignant pleural mesothelioma. *Cancer Treatment Reviews*.

[10] Hulleman J. D. (2016). Malattia Leventinese/Doyne honeycomb retinal dystrophy: similarities to age-related macular degeneration and potential therapies. *Advances in Experimental Medicine and Biology*.

[11] Xu S., Yang Y., Sun Y. B., Wang H. Y., Sun C. B., Zhang X. (2014). Role of fibulin-3 in lung cancer: in vivo and in vitro analyses. *Oncology Reports*.

[12] Sadr-Nabavi A., Ramser J., Volkmann J. (2009). Decreased expression of angiogenesis antagonist EFEMP1 in sporadic breast cancer is caused by aberrant promoter methylation and points to an impact of EFEMP1 as molecular biomarker. *International Journal of Cancer*.

[13] Luo R., Zhang M., Liu L., Lu S., Zhang C. Z., Yun J. (2013). Decrease of fibulin-3 in hepatocellular carcinoma indicates poor prognosis. *PLoS One*.

[14] Pass H. I., Liu Z., Wali A. (2004). Gene expression profiles predict survival and progression of pleural mesothelioma. *Clinical Cancer Research*.

[15] Tsim S., Kelly C., Alexander L. (2016). Diagnostic and prognostic biomarkers in the rational assessment of mesothelioma (DIAPHRAGM) study: protocol of a prospective, multicentre, observational study. *BMJ Open*.

[16] Pass H. I., Levin S. M., Harbut M. R. (2012). Fibulin-3 as a blood and effusion biomarker for pleural mesothelioma. *The New England Journal of Medicine*.

[17] Kirschner M. B., Pulford E., Hoda M. A. (2015). Fibulin-3 levels in malignant pleural mesothelioma are associated with prognosis but not diagnosis. *British Journal of Cancer*.

[18] Ying S., Jiang Z., He X. (2017). Serum HMGB1 as a potential biomarker for patients with asbestos-related diseases. *Disease Markers*.

[19] American Thoracic Society (2004). Diagnosis and initial management of nonmalignant diseases related to asbestos. *American Journal of Respiratory and Critical Care Medicine*.

[20] Husain A. N., Colby T., Ordonez N. (2013). Guidelines for pathologic diagnosis of malignant mesothelioma: 2012 update of the consensus statement from the International Mesothelioma Interest Group. *Archives of Pathology & Laboratory Medicine*.

[21] Yu M., Zhang Y., Jiang Z. (2015). Mesothelin (MSLN) methylation and soluble mesothelin-related protein levels in a Chinese asbestos-exposed population. *Environmental Health and Preventive Medicine*.

[22] Ortega-Guerrero M. A., Carrasco-Nunez G., Barragan-Campos H., Ortega M. R. (2015). High incidence of lung cancer and malignant mesothelioma linked to erionite fibre exposure in a rural community in Central Mexico. *Occupational and Environmental Medicine*.

[23] Prazakova S., Thomas P. S., Sandrini A., Yates D. H. (2014). Asbestos and the lung in the 21st century: an update. *The Clinical Respiratory Journal*.

[24] Park E. K., Yates D. H., Creaney J., Thomas P. S., Robinson B. W., Johnson A. R. (2012). Association of biomarker levels with severity of asbestos-related diseases. *Safety and Health at Work*.

[25] Pass H. I., Lott D., Lonardo F. (2005). Asbestos exposure, pleural mesothelioma, and serum osteopontin levels. *The New England Journal of Medicine*.

[26] Cui A., Jin X. G., Zhai K., Tong Z. H., Shi H. Z. (2014). Diagnostic values of soluble mesothelin-related peptides for malignant pleural mesothelioma: updated meta-analysis. *BMJ Open*.

[27] Cristaudo A., Bonotti A., Simonini S. (2011). Combined serum mesothelin and plasma osteopontin measurements in malignant pleural mesothelioma. *Journal of Thoracic Oncology*.

[28] Hollevoet K., Nackaerts K., Thimpont J. (2010). Diagnostic performance of soluble mesothelin and megakaryocyte potentiating factor in mesothelioma. *American Journal of Respiratory and Critical Care Medicine*.

[29] Corradi M., Goldoni M., Alinovi R. (2013). YKL-40 and mesothelin in the blood of patients with malignant mesothelioma, lung cancer and asbestosis. *Anticancer Research*.

[30] Lacourt A., Rolland P., Gramond C. (2010). Attributable risk in men in two French case-control studies on mesothelioma and asbestos. *European Journal of Epidemiology*.

[31] Hu Y., Pioli P. D., Siegel E. (2011). EFEMP1 suppresses malignant glioma growth and exerts its action within the tumor extracellular compartment. *Molecular Cancer*.

[32] Obaya A. J., Rua S., Moncada-Pazos A., Cal S. (2012). The dual role of fibulins in tumorigenesis. *Cancer Letters*.

[33] Tian H., Liu J., Chen J., Gatza M. L., Blobe G. C. (2015). Fibulin-3 is a novel TGF-*β* pathway inhibitor in the breast cancer microenvironment. *Oncogene*.

[34] Kim I. G., Kim S. Y., Choi S. I., Lee J. H., Kim K. C., Cho E. W. (2014). Fibulin-3-mediated inhibition of epithelial-to-mesenchymal transition and self-renewal of ALDH+ lung cancer stem cells through IGF1R signaling. *Oncogene*.

[35] Chen X., Meng J., Yue W. (2014). Fibulin-3 suppresses Wnt/β-catenin signaling and lung cancer invasion. *Carcinogenesis*.

[36] Kovac V., Dodic-Fikfak M., Arneric N., Dolzan V., Franko A. (2015). Fibulin-3 as a biomarker of response to treatment in malignant mesothelioma. *Radiology and Oncology*.

[37] Creaney J., Dick I. M., Meniawy T. M. (2014). Comparison of fibulin-3 and mesothelin as markers in malignant mesothelioma. *Thorax*.

[38] Park E. K., Takahashi K., Hoshuyama T. (2011). Global magnitude of reported and unreported mesothelioma. *Environmental Health Perspectives*.

